# Serial testing of latent tuberculosis infection in patients with diabetes mellitus using interferon-gamma release assay, tuberculin skin test, and creation tuberculin skin test

**DOI:** 10.3389/fpubh.2022.1025550

**Published:** 2022-12-01

**Authors:** Yijun He, Xuefang Cao, Tonglei Guo, Yongpeng He, Ying Du, Haoran Zhang, Boxuan Feng, Jiang Du, Bin Zhang, Kun Wang, Jiaoxia Yan, Dakuan Wang, Zisen Liu, Shouguo Pan, Henan Xin, Lei Gao

**Affiliations:** ^1^NHC Key Laboratory of Systems Biology of Pathogens, Institute of Pathogen Biology and Center for Tuberculosis Research, Chinese Academy of Medical Sciences and Peking Union Medical College, Beijing, China; ^2^Center for Diseases Control and Prevention of Zhongmu, Zhengzhou, China

**Keywords:** latent tuberculosis infection, diabetes mellitus, interferon-gamma release assay, tuberculin skin test, creation tuberculin skin test

## Abstract

**Background:**

Diabetes mellitus (DM) patients with latent tuberculosis infection (LTBI) have an increased risk of developing active tuberculosis (TB) due to impaired immunity. The performance of currently available immune response-based assays for identification of TB infection had been rarely evaluated in patients with type 2 DM (T2DM) in China.

**Methods:**

A prospective study was conducted to investigate the status of LTBI in patients with confirmed T2DM. At the baseline survey, the prevalence of LTBI was tested using interferon-gamma release assay (IGRA), tuberculin skin test (TST) and creation tuberculin skin test (C-TST) in parallel. After a 3-month interval, the participants were retested by the three assays to estimate their performance in the serial testing.

**Results:**

A total of 404 participants with T2DM were included in the study. At baseline, after excluding active TB, the prevalence of LTBI identified by TST (≥ 10 mm), C-TST (≥ 5 mm) and IGRA (≥ 0.35 IU/ml) were 9.65% (39/404), 10.40% (42/404) and 14.85% (60/404), respectively. The concordance of TST and C-TST results with IGRA results was 86.39% (349/404) and 92.08% (372/404) with a Kappa coefficient of 0.37 [95% confidence interval (CI): 0.24– 0.50] and 0.64 (95% CI: 0.53– 0.76), respectively. After a 3-month interval, the continuous results of TST, C-TST and IGRA were observed to be increased with testing conversion for 50, 26 and 27 patients, respectively. For TST and C-TST conversions, the distribution of their quantitative results in serial tests varied significantly when further classified by baseline IGRA dichotomous results.

**Conclusion:**

In studied patients with T2DM, C-TST showed higher consistency with IGRA as compared to TST. The present of conversion observed in serial testing suggested that boosting effect of skin testing should be considered for identify of LTBI in patients with T2DM.

## Introduction

As an ancient infectious disease, tuberculosis (TB) is still one of the leading causes of morbidity and mortality worldwide (1). About a quarter of the world's population is infected with *Mycobacterium tuberculosis* (MTB), and people with latent tuberculosis infection (LTBI) are at risk of progressing to active TB disease, especially among those with comorbidity, such as diabetes mellitus (DM) (1, 2). A systematic review has revealed that DM can impair host immunity and increase the risk of active TB approximately threefold (2). In China, the prevalence of DM increased from 10.9% in 2013 to 12.4% in 2018 (3). The increasing prevalence of type 2 DM (T2DM), especially in TB high-burden countries, has therefore generated concerns that this double epidemic could undermine the TB control effort (4). Currently, tuberculosis preventive treatment (TPT) has been reported to reduce the risk of active TB development with efficacy ranging from 60% to 90% and recommended for LTBI with a high risk of progressing to active TB, such as human immunodeficiency virus infections and household contacts (5–7). However, due to the lack of strong evidence, systematic testing for LTBI and initialing TPT is not currently recommended in T2DM patients (8). More evidence is needed to explore suitable intervention strategies for T2DM patients through clinical trials. Evaluation of the prevalence of LTBI in T2DM patients accurately was the prerequisite for administrating of TPT subsequently.

Currently, interferon-gamma (IFN-γ) release assay (IGRA) and tuberculin skin test (TST) are the two TB infection diagnosis tools recommended by the world health organization (8). The TST is widely used and inexpensive, but it has poor specificity in populations vaccinated with bacillus Calmette-Guerin (BCG), is subject to cross-reactivity with environmental non-tuberculosis mycobacteria (NTM), and has poor sensitivity in immunocompromised persons (9). IGRA has been recommended for testing TB infection and showed better specificity than the TST, especially in BCG vaccinated populations, however, the test is expensive and requires a well-established laboratory (7, 10). To combine the advantages of the high specificity of IGRA and ease of TST operation, creation tuberculin skin test (C-TST) had been developed to measure the cell-mediated immunological response to MTB-specific antigens in recent years (11). C-TST had not been systematically evaluated in various populations, and along with IGRA and TST, all the three detection methods are based on immunological response. Due to T2DM can impair host immune function (12, 13), whether the performance of TST, IGRA and C-TST would be influenced in T2DM patients had been rarely studied. The current study detected the prevalence of LTBI in registered T2DM patients from rural China using three assays in parallel twice (baseline and 3 months later), aiming to evaluate the performance of IGRA, TST and C-TST in serial testing among T2DM patients.

## Methods

### Study design

The study aimed to evaluate the performance of IGRA, TST and C-TST and clarify the LTBI burden in studied T2DM patients. The baseline survey was conducted in November 2021, in which T2DM patients were screened for LTBI using IGRA, TST and C-TST in parallel. Considering the estimated interval between infection and detectable reactivity (referred to as the window period of immunological tests) is 8–10 weeks (14, 15), retesting was conducted for baseline participants by the three assays after a 3-month interval.

### Study participants

The target population of this study was the rural community residents aged 18–65 years who had been diagnosed with T2DM according to the standards of medical care for T2DM in China (2019) (16) and managed through the chronic disease management system of Zhongmu County, Henan Province, where the epidemic of TB and T2DM is similar to the average in China. During the recruitment, the diagnosis of T2DM was validated by investigating their blood glucose and medication: currently receiving antidiabetic drug, or not receiving antidiabetic drug but the level of fasting plasma glucose (FPG) ≥ 7.0 mmol/L and hemoglobin A1c (HbA1c) ≥ 6.5%. In addition, the included participants should fulfill the following criteria: household registration or residence permit for that village; continuous residence at the study site for ≥ 6 months over the past year; ability to complete the investigations and tests during the study duration, and provision of voluntary written informed consent. The exclusion criteria: current active TB; self-reported history of TB; pregnant or lactating women or women preparing for pregnancy.

### Procedures

For each enrolled study participant, socio-demographic information was collected by a standardized questionnaire administered by trained interviewers. Data were collected on age, gender, educational level, household per capita income, self-reported history of close contact with TB patients, smoking status, and alcohol consumption within the past 12 months. Digital chest radiography was performed on each study participant to exclude individuals with active TB or suspect TB. Height and weight were measured as well.

Before the administration of TST (Purified Protein Derivative of Tuberculin; Xiang Rui, Beijing, China) and C-TST (Recombinant Mycobacterium Tuberculosis Fusion Protein; Zhifei Longcom Biologic Pharmacy Company, Anhui, China), 4 ml venous blood samples were collected in a single tube for IGRA (QuantiFERON-TB Gold Plus; Qiagen; Germantown, MD, USA). Then, participants immediately underwent skin testing of 0.1 mL (5IU) purified protein derivative of tuberculin on the volar surface of the right forearm and 0.1 mL (5U) recombinant mycobacterium tuberculosis fusion protein on the left forearm as self-control, with a 5-minute interval between TST and C-TST. TST and C-TST were injected according to the Mantoux technique. Two trained study personnel independently read induration and erythema reactions at TST and C-TST injection sites after 48–72 h of placement and recorded in millimeters (mm). A positive TST result was defined as an average diameter of induration ≥ 10 mm. TST conversion was defined as the average diameter of induration ≥ 10 mm for those with baseline survey < 5 mm or increased ≥ 10 mm when the baseline survey result was between 5 and 10 mm. The results of the C-TST were considered positive when the average diameter of induration or erythema was ≥ 5 mm (17). C-TST conversion was defined as the average diameter of induration and erythema increased from <5 mm at baseline survey to induration or erythema ≥ 5 mm at follow-up survey. Information on adverse events was self-reported by participants. IGRA was performed as recommended by the manufacturer using a cut-off value of ≥ 0.35 IU/ml. IGRA conversion was defined as the IFN-γ releasing level of TB antigen-Nil (TBAg-Nil) increased from < 0.35 IU/ml at baseline survey to ≥ 0.35 IU/ml at follow-up survey.

### Statistical analysis

Questionnaire data, physical examination data (height, weight, TST induration, C-TST induration and erythema), and laboratory results (IGRA and blood biochemical examination) were double entered using EpiData 3.1 software. Continuous variables were presented using median value and interquartile range (IQR). Body mass index (BMI) was calculated as weight over height squared (kg/m^2^). Pearson's Chi-squared test was used to compare the distribution of categorical variables in the study participants. Wilcoxon matched-pairs signed-rank test was used to compare the differences in TST induration, C-TST induration/ erythema and IFN-γ levels (TB1-Nil, TB2-Nil) between baseline and 3-month follow-up survey. Agreement between the three assays was calculated and presented with concordance and Cohen's kappa coefficient. Sensitivity analysis were conducted for TST using a cut-off value of 5 mm as positive. Data were analyzed using Statistical Analysis System (SAS 9.4; SAS Institute Inc, NC, USA). *P*-values below 0.05 were considered statistically significant.

## Results

### Baseline characteristics of study participants

There were 468 eligible registered rural community T2DM patients aged 18–65 years who participated in the baseline survey. Among them, 87.6% (410/468) completed the 3-month follow-up survey. After excluding 6 participants with missing data, 404 participants were included in the final analysis. Overall, the median age of the participants was 57 years (IQR: 53–60), and 61.88% (250/404) were female. Nearly 60% (58.91%, 238/404) of the participants had typical symptoms of T2DM and 10% (11.14%, 45/404) of the participants had T2DM complications. The baseline median level of FPG and HbA1c were 9.03 mmol/L (IQR: 7.27–11.73) and 9.80% (IQR: 8.50–11.60), respectively (Table 1).

**Table 1:** Characteristics of the study participants.

**Variables**	***n* (%)**
Total	404 (100.00)
Sex	
Male	154 (38.12)
Female	250 (61.88)
Age (years)	
< 50	48 (11.88)
50–59	240 (59.41)
≥ 60	116 (28.71)
Highest education level	
Lower than primary school	92 (22.77)
Primary school	135 (33.42)
Middle school	132 (32.67)
High school or higher	45 (11.14)
Household per capita income (RMB)	
< 6000	197 (48.76)
≥ 6000	207 (51.24)
BMI (kg/m^2^)	
18− < 24	87 (21.53)
24− < 28	189 (46.78)
≥ 28	128 (31.68)
Smoking status	
Never smoker	307 (75.99)
Former smoker	33 (8.17)
Current smoker	64 (15.84)
Alcohol consumption (within the past 12 months)	
No	315 (77.97)
Yes	89 (22.03)
History of close contact with TB patients	
No	394 (97.52)
Yes	10 (2.48)
DM complications^†^	
Absent	359 (88.86)
Present	45 (11.14)
Accepting regular treatment of DM^&^	
No	58 (14.36)
Yes	346 (85.64)
The level of HbA1c at baseline (%)	
< 7.0	23 (5.69)
≥ 7.0	381 (94.31)
The level of FPG at baseline (mmol/L)	
< 7.0	83 (20.54)
≥ 7.0	321 (79.46)

BMI, Body mass index; DM, Diabetes mellitus; FPG, Fasting plasma glucose; HbA1c, Hemoglobin A1c; TB, Tuberculosis.

† Macrovascular complications (cardiovascular disease) and microvascular complications (such as diabetic kidney disease, diabetic retinopathy, neuropathy, etc.) were investigated.

& Using national guidelines recommended regimens with good adherence for DM treatment and monitoring blood glucose level periodically.

### The prevalence of LTBI identified by three assays and their concordance

At baseline, after excluding active TB, the prevalence of LTBI identified by TST (≥10 mm), C-TST (≥ 5 mm) and IGRA (≥ 0.35 IU/ml) were 9.65% (39/404), 10.40% (42/404) and 14.85% (60/404), respectively. After a 3-month interval, the results of TST, C-TST and IGRA were converted for 50, 26 and 27 patients, respectively, with the corresponding prevalence of LTBI of 21.04% (85/404), 15.84% (64/404) and 19.55% (79/404). Compared to the baseline survey, the positivity rate was significantly higher for all three assays at the 3-month follow-up survey (*p* < 0.001) (Table 2).

**Table 2:** The positivity of IGRA, TST and C-TST at the baseline and 3-month follow-up survey.

	**TST positivity (≥10 mm), *n* (%)**	**C-TST positivity (≥5 mm), *n* (%)**	**IGRA positivity (0.35 IU/mL), *n* (%)**
**At baseline (*****N =*** **404)**	39 (9.65)	42 (10.40)	60 (14.85)
At 3-month follow-up survey^‡^ (*N =* 404)	85 (21.04)	64 (15.84)	79 (19.55)
Converted	50 (58.82)^*^	26 (40.63)^&^	27 (34.18)^**†**^
Persistent positive^**¶**^	35 (41.18)	38 (59.38)	52 (65.82)
**p for** ***χ**^2^* **test**	< 0.001	< 0.001	< 0.001

C-TST, Creation tuberculin skin test; IGRA, Interferon-gamma release assay; TST, Tuberculin skin test.

‡ The definition of being positive at the 3-month follow-up survey was being persistent positive or being converted.

* TST conversion was defined as the average diameter of induration ≥ 10 mm for those with baseline survey < 5 mm, or increased ≥ 10 mm when the baseline survey result was between 5 and 10 mm.

& C-TST conversion was defined as the average diameter of induration and erythema increased from < 5mm at baseline survey to induration or erythema ≥ 5mm at follow-up survey.

† IGRA conversion was defined as the IFN-γ releasing level of TB antigen-Nil (TBAg-Nil) increased from < 0.35 IU/ml at baseline survey to ≥ 0.35 IU/ml at follow-up survey.

¶ Persistent positive was defined as both positive for baseline and follow-up surveys according to the baseline positivity cutoff value.

At baseline, the concordance of TST and C-TST results with IGRA results was 86.39% (349/404) and 92.08% (372/404) with a Kappa coefficient of 0.37 [95% confidence interval (CI): 0.24–0.50] and 0.64 (95% CI: 0.53–0.76), respectively. At the 3-month follow-up survey, with the addition of convertors, the concordance of TST and C-TST results with baseline IGRA results was 85.40% (345/404) and 94.06% (380/404) with a Kappa coefficient of 0.51 (95% CI: 0.40–0.62) and 0.77 (95% CI: 0.68–0.86) (Table 3). When using 5mm as a cut-off value for TST, the results did not change remarkably as seen in Supplementary Table 1.

**Table 3:** Agreement between baseline IGRA results and TST/C-TST results at the baseline and 3-month follow-up survey.

**Agreement between IGRA results and TST/C-TST results at baseline**						
Definition of positive	TST-/IGRA- n (%)	TST+/IGRA+ n (%)	TST-/IGRA+ n (%)	TST+/IGRA- n (%)	Kappa (95% CI)	Concordant (%)
≥ 10 mm	327 (80.94)	22 (5.45)	38 (9.41)	17 (4.21)	0.37 (0.24- 0.50)	86.39
Definition of positive	C-TST -/IGRA- n (%)	C-TST +/IGRA+ n (%)	C-TST -/IGRA+ n (%)	C-TST +/IGRA- n (%)	Kappa (95% CI)	Concordant (%)
≥ 5 mm	337 (83.42)	35 (8.66)	25 (6.19)	7 (1.73)	0.64 (0.53- 0.76)	92.08
**Agreement between baseline IGRA results and TST/C-TST results at 3-month follow-up survey**						
Definition of positive	TST-/IGRA- n (%)	TST+/IGRA+ n (%)	TST-/IGRA+ n (%)	TST+/IGRA-n (%)	Kappa (95% CI)	Concordant (%)
converted or persistent positive (≥ 10 mm)	302 (74.75)	43 (10.64)	17 (4.21)	42 (10.40)	0.51 (0.40-0.62)	85.40
Definition of positive	C-TST -/IGRA- n (%)	C-TST +/IGRA+ n (%)	C-TST -/IGRA+ n (%)	C-TST +/IGRA-n (%)	Kappa (95% CI)	Concordant (%)
converted or persistent positive (≥ 5 mm)	330 (81.68)	50 (12.38)	10 (2.48)	14 (3.47)	0.77 (0.68-0.86)	94.06

CI, Confidence interval; C-TST, Creation tuberculin skin test; IGRA, Interferon-gamma release assay; TST, Tuberculin skin test; A cutoff value of 0.35 IU/mL was used for IGRA.

### Serial qualitative and quantitative results of TST and C-TST

**Figure 1:**
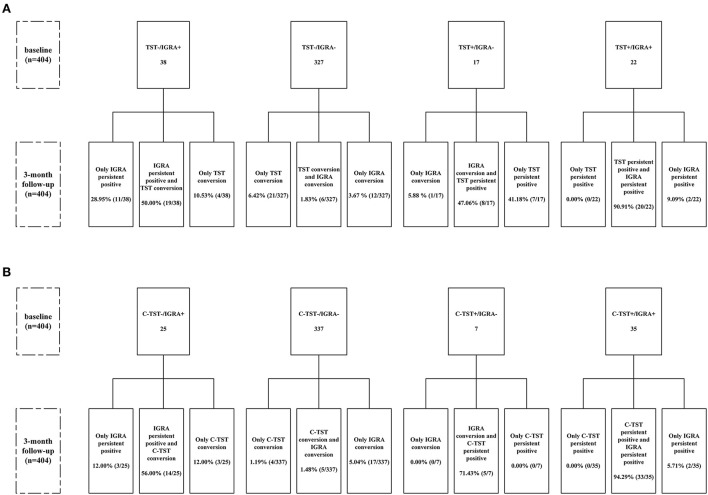
Flow chart of serial testing of TST and C-TST according to IGRA results. **(A)** Stratified analysis according to baseline results of IGRA and TST (using cutoff value of 10 mm); **(B)** Stratified analysis according to baseline results of IGRA and C-TST. C-TST, Creation tuberculin skin test; IGRA, Interferon-gamma release assay; TST, Tuberculin skin test. TST conversion was defined as the average diameter of induration ≥ 10 mm for those with baseline survey < 5 mm, or increased ≥ 10 mm when the baseline survey result was between 5 and 10 mm. C-TST conversion was defined as the average diameter of induration and erythema increased from < 5mm at baseline survey to induration or erythema ≥ 5mm at follow-up survey. IGRA conversion was defined as the IFN-γ releasing level of TB antigen-Nil (TBAg-Nil) increased from < 0.35 IU/ml at baseline survey to ≥ 0.35 IU/ml at follow-up survey. Persistent positive was defined as both positive for baseline and follow-up surveys according to the baseline positivity cutoff value.

Serial qualitative results of IGRA, TST and C-TST at the baseline and 3-month follow-up survey were shown in Figure 1. The 404 participants were divided into 4 subgroups based on the baseline results of IGRA vs. TST or IGRA vs. C-TST. Conversions were frequently observed for all three assays after a 3-month interval. The conversions rate of TST and C-TST was 60.53% (23/38) and 68.00% (17/25), respectively in the baseline IGRA-positive group and 8.26% (27/327) and 2.67% (9/337) in the baseline IGRA-negative group, respectively. When using 5mm as a cut-off value for TST, the results did not change remarkably as shown in Supplementary Figure 1.

As shown in Table 4, participants were divided into two subgroups according to the testing results at baseline and 3-month follow-up survey. Compared with baseline results, the median level of quantitative results of IGRA, TST and C-TST increased significantly after a 3-month interval, no matter for conversion subgroup or persistence subgroup.

**Table 4:** Quantitative results of TST, C-TST and IGRA at the baseline and 3-month follow-up survey.

**Subgroup**	**TST induration mm,median (IQR)**				**C-TST induration/ erythema mm, median (IQR)**				**IGRA**						
									**TB1-Nil IU/ml, median (IQR)**				**TB2-Nil IU/ml, median (IQR)**		
	***N***	**Baseline**	**3-month follow-up**	***P***	***N***	**Baseline**	**3-month follow-up**	***P***	***N***	**Baseline**	**3-month follow-up**	***P***	**Baseline**	**3-month follow-up**	***P***
Baseline -/follow-up +	50	0.00 (0.00-0.00)	13.50 (11.50-17.00)	< 0.001	26	0.00 (0.00-0.00)	20.00 (17.00-24.00)	< 0.001	27	0.13 (0.03-0.23)	0.50 (0.29-1.41)	< 0.001	0.16 (0.05-0.24)	0.62 (0.36-1.47)	< 0.001
Baseline +/follow-up +	35	15.00 (11.50-19.50)	17.00 (16.00-20.00)	0.043	38	18.00 (14.00-23.50)	24.00 (20.00-31.00)	< 0.001	52	1.24 (0.60-2.68)	2.10 (1.15-4.12)	< 0.001	1.12 (0.64-2.78)	2.33 (1.14-3.80)	< 0.001

C-TST, Creation tuberculin skin test; IGRA, Interferon-gamma release assay; IQR, Interquartile range; TST, Tuberculin skin test; The differences were tested by Wilcoxon matched-pairs signed-rank test.

Serial quantitative results of TST and C-TST in converted subjects classified by baseline IGRA results were presented in Table 5. The median level of TST and C-TST increased from 0 mm to 13.5 mm and 0 mm to 20 mm respectively in baseline IGRA positive subgroup. At the 3-month follow-up survey, for 27 TST conversions with baseline IGRA negative results, the median level of TB1-Nil and TB2-Nil were 0.01 IU/ml (IQR: 0.00–0.20) and 0.03 IU/ml (IQR: 0.00–0.22), respectively. For 9 C-TST conversions with baseline IGRA negative results, the median level of TB1-Nil and TB2-Nil was 0.43 IU/ml (IQR: 0.08–0.76) and 0.62 IU/ml (IQR: 0.06–0.72), respectively.

**Table 5:** Quantitative results of TST, C-TST and IGRA in skin testing converted subjects.

TST conversion^*^(*N =* 50)	**Baseline IGRA**+ **(*****N =*** **23) median (IQR)**			**Baseline IGRA- (*****N =*** **27) median (IQR)**		
	**Baseline**	**3-month follow-up**	***p***	**Baseline**	**3-month follow-up**	***p***
TST induration, mm	0.00 (0.00–0.00)	13.50 (11.00–20.00)	< 0.001	0.00 (0.00–0.00)	13.50 (11.50–16.00)	< 0.001
IGRA TB1-Nil, IU/ml	0.85 (0.49–1.44)	1.33 (0.43–3.70)	0.020	0.01 (0.00–0.08)	0.01 (0.00–0.20)	0.024
IGRA TB2-Nil, IU/ml	0.81 (0.51–1.46)	1.43 (0.73–3.49)	0.017	0.02 (0.00–0.08)	0.03 (0.00–0.22)	0.004
**C-TST conversion**^&^ **(*****N** =* **26)**	**Baseline IGRA**+ **(*****N** =* **17) median (IQR)**			**Baseline IGRA– (*****N** =* **9) median (IQR)**		
	Baseline	**3–month follow–up**	***p***	**Baseline**	**3–month follow–up**	***p***
C-TST induration/ erythema, mm	0.00 (0.00–0.00)	20.00 (17.50–23.00)	< 0.001	0.00 (0.00–0.00)	18.00 (10.00–24.00)	0.004
IGRA TB1-Nil, IU/ml	0.68 (0.49–2.01)	1.45 (0.97–2.51)	0.132	0.04 (0.01–0.09)	0.43 (0.08–0.76)	0.016
IGRA TB2-Nil, IU/ml	0.72 (0.55–1.78)	1.45 (0.86–2.28)	0.109	0.02 (0.01–0.08)	0.62 (0.06–0.72)	0.016

C-TST, Creation tuberculin skin test; IGRA, Interferon-gamma release assay; IQR, Interquartile range; TST, Tuberculin skin test; The differences were tested by Wilcoxon matched-pairs signed-rank test.

* TST conversion was defined as the average diameter of induration ≥10 mm for those with baseline survey < 5 mm, or increased ≥ 10 mm when the baseline survey result was between 5 and 10 mm.

& C-TST conversion was defined as the average diameter of induration and erythema increased from < 5mm at baseline survey to induration or erythema ≥ 5mm at follow-up survey.

The level of FPG in the baseline positive and conversions groups of TST and C-TST were shown in Supplementary Figure 2. For TST, the median level of FPG in the baseline positive (*N* = 39) and conversion (*N* = 50) group was 7.74 mmol/L (IQR: 6.70–9.75) and 9.35 mmol/L (IQR: 7.02–12.64) (*p* = 0.058), respectively. For C-TST, the median level of FPG in the baseline positive (*N* = 42) and conversion (*N* = 26) group was 8.11 mmol/L (IQR: 6.70-10.12) and 8.71 mmol/L (IQR: 7.26–10.42) (*p* = 0.464), respectively.

### Self-reported adverse events of TST and C-TST

Three (0.74%) of the 404 T2DM patients reported adverse events, including one patient with pain at the injection site of C-TST, one with itching at the injection site of TST, and one with fever. No serious adverse events were reported.

## Conclusion

In conclusion, the prevalence of LTBI identified by IGRA is higher than TST or C-TST and the performance of all three assays was influenced due to impaired immunity in studied T2DM patients. Repeat tests might retrieve parts of infections and improve the sensitivity of skin tests in immunocompromised people through boosting effect, but our findings need further exploration and verification.

## Discussion

To our knowledge, this is the first study to evaluate the performance of IGRA, TST and C-TST in parallel in T2DM patients using serial testing. In this target population for TPT, we found that IGRA showed the highest positivity rate of 14.85% (60/404) compared with TST (9.65%, 39/404) and C-TST (10.40%, 42/404) at the baseline survey, and C-TST showed higher consistency with IGRA as compared to TST. Conversions occurred for all three tests after a 3-month interval and increased the positivity rate statistically significant, which indicated boosting effect existed for skin tests and IGRA results might be affected by previous skin tests as well.

Due to the increased risk of developing TB among people with T2DM and the large number of patients with combined TB and T2DM (2, 18), it is no doubt that people with T2DM should be targeted with TPT. However, in the absence of clinical trial data, guidelines do not recommend LTBI screening and TPT among them currently (8, 19). Before initialing TPT, identifying LTBI accurately was one of the challenges faced as current available LTBI diagnostic tools were based on the immune response. Previous studies have found that poor glycemic control may lead to a blunted immune response (12, 13). Neutrophil chemotaxis and oxidative killing potential were reduced in DM patients compared to nondiabetic controls, and leukocyte bactericidal activity was reduced, especially in patients with poor glycemic control (2). In our study, although most of the patients received regular treatment for T2DM, their blood sugar was not well controlled. The prevalence of LTBI at the baseline survey identified by TST, C-TST and IGRA in T2DM patients with a median age of 57 years (IQR: 53–60) was 9.65% (39/404), 10.40% (42/404) and 14.85% (60/404), which were remarkably lower than the general population with median age of 59 years (IQR: 54–64) from the same study site with an IGRA positivity of 20.79% (4259/20486) (20). In comparison to our study, a meta-analysis involving 38263 participants from 13 studies reported DM was associated with a small but statistically significant risk for LTBI (pooled odds ratio: 1.18, 95% CI: 1.06–1.30) (4). The different characteristic of study population (household contacts, immigrants, and immunosuppressed populations) and DM diagnosis criteria (majority of them were unclear or self-report) might partly explain the discrepancy between our study and the meta-analysis.

A high proportion of conversion was observed for all three assays. For the serial testing, whether such conversion represents new infection, non-specific variation due to potential influence from initial tests, or just test reproducibility because of its intradermal application is still unclear (21, 22). In our study, one patient with a history of close contact with TB patients before enrollment underwent TST conversion after a 3-month interval. Although none of the participants reported close contact history within 3 months, new infections arising from close contacts before and after enrolment could not be fully excluded due to the testing window period (14, 15). More important, previous studies have reported that the boosting effect diminishes until months or even occurs after 1 year (23, 24). Previous guidelines have suggested that the probability of LTBI was greatest when both IGRA and TST were positive, and the probability of LTBI was least when both IGRA and TST were negative (25). Therefore, the conclusions about whether boosting effects occurred need to be related to the initial status of the participants. Combining the results of IGRA, TST and C-TST pre-and-post 3-month intervals together could help us clarify the possible reasons.

For TST conversion, the median levels of IFN-γ in the baseline IGRA-positive group were 1.33 IU/ml for TB1-Nil and 1.43 IU/ml for TB2-Nil, respectively. In contrast, the median levels of IFN-γ in the baseline IGRA-negative group were 0.01 IU/ml for TB1-Nil and 0.03 IU/ml for TB2-Nil, respectively. Therefore, although the quantitative IFN-γ level in the baseline IGRA-negative group was increased significantly after a 3-month interval, this change might not be defined as a conversion. The participants who were TST-negative and IGRA-positive at baseline had higher levels of absolute IFN-γ responses after a 3-month interval and were more likely to undergo conversion. Our results may suggest that participants with TST conversion in the baseline IGRA-positive group are more inclined to the true LTBI status and that the occurrence of conversion is mainly an effect of boosting. In contrast, the baseline IGRA-negative group was more inclined to a false-positive conversion caused by tuberculin injection from initial tests. In addition, previous meta-analysis showed the effect of TST on subsequent IGRA was apparent only after a few days and waned after 3 months (26), the increased median level of IFN-γ after 3 months observed in our study, especially in initial IGRA positives, consistently reminded us to pay attention to the time interval when applying IGRA and skin tests jointly. Of course, the results should be explained with caution because of the lack of a gold standard for LTBI.

C-TST using only two specific MTB antigens [early secreted antigenic target 6 (ESAT-6) and culture filtrate protein 10 (CFP-10)] instead of the tuberculin solution has recently been developed (27). Although previous studies have shown no indication of cross-reactivity between TST and C-TST, it is well known that purified protein derivative preparations contain immunologically active ESAT-6 and CFP-10 that might boost C-TST responses (28–30). In addition, repeated skin testing with a recombinant dimer of ESAT-6 may also cause a low frequency of sensitization reactions (31, 32). Unlike TST, the median level of IFN-γ in the baseline IGRA-negative group of C-TST converters was increased significantly after a 3-month interval and above the threshold of 0.35 IU/ml. It indicated participants with C-TST conversion in the baseline IGRA-negative group are more inclined to the true LTBI status. T2DM patients may have a false-negative TST or C-TST due to immunosuppression (12, 33). However, the use of the booster effect to increase the detection of LTBI had been recommended in different populations, including immunosuppressed patients as well as healthy population (34–36). Based on the current findings, it is, therefore, reasonable to speculate that serial testing of LTBI using C-TST may be a potentially effective strategy to exclude false-negative effects in immunosuppressed populations. However, more studies are needed to explore appropriate strategies for LTBI screening among T2DM patients using C-TST.

When interpreting the results of the present study, there are limitations. First, our study participants might not completely represent the local T2DM patients with respect to the distribution of age and gender. The degree of concern to their own health and whether being available to participate in the survey may lead to potential bias. Second, the number of convertors was too small to identify potential risk factors associated with the acquisition of TB infection among T2DM patients. Multicenter studies with a large sample size are needed. In addition, the conversion, especially more than one type of test in single patient, might indicate increased risk of active TB development. Due to the limited sample size, we cannot obtain valuable results at present, further observation would be continued to track the development of active TB in the study participants. Third, all participants completed TST and C-TST screening in parallel as self-control. We were unable to differentiate boosting effect belongs to TST or C-TST. Fourth, a single cutoff value of 0.35 IU·mL^−1^ was used for IGRA to define conversion in our study. Therefore, reproducibility is an important consideration that makes it challenging to use a single cutoff value to distinguish between positive and negative results with one-time testing and to define conversion in individuals undergoing serial testing. Fifth, although study personnel administering and reading the skin tests were properly trained, measurement of the response—the size of a skin induration or erythema—is subject to misclassification.

## Data availability statement

The original contributions presented in the study are included in the article/Supplementary material, further inquiries can be directed to the corresponding authors.

## Ethics statement

The studies involving human participants were reviewed and approved by the Ethics Committee of the Institute of Pathogen Biology, Chinese Academy of Medical Sciences (Beijing, China) (IPB-2021-09). The patients/participants provided their written informed consent to participate in this study.

## Author contributions

LG designed the study. ZL and SP coordinated the study implementation and management. YiH, YD, XC, BF, TG, YoH, and JY were responsible for laboratory testing. HZ, BZ, DW, KW, and JD contributed to field investigation and quality control. YiH and HX did data management and data analysis. YiH, HX, and LG wrote the report. All authors contributed to review and revision and have seen and approved the final version of manuscript.

## Funding

This work was supported by the CAMS Innovation Fund for Medical Sciences (2021-I2M-1-037), the Fundamental Research Funds for the Central Universities (3332021092). They did not involve in trial design, patient recruitment, data collection, analyses, and interpretation or any aspect pertinent to the study.

## Conflict of interest

The authors declare that the research was conducted in the absence of any commercial or financial relationships that could be construed as a potential conflict of interest.

## Publisher's note

All claims expressed in this article are solely those of the authors and do not necessarily represent those of their affiliated organizations, or those of the publisher, the editors and the reviewers. Any product that may be evaluated in this article, or claim that may be made by its manufacturer, is not guaranteed or endorsed by the publisher.
