# Supplementary material for: Serial testing of latent tuberculosis infection in patients with diabetes mellitus using interferon-gamma release assay, tuberculin skin test, and creation tuberculin skin test
Source: Front Public Health. 2022 Dec 1;10:1025550. doi: 10.3389/fpubh.2022.1025550 (PMC9754324; doi:10.3389/fpubh.2022.1025550)
Supplement: Supplementary file 1 [file Data_Sheet_1.docx]

**Supplementary table 1**

**Agreement between baseline IGRA results and TST results at the baseline and 3-month follow-up survey**

| **Agreement between IGRA results and TST results at baseline** | | | | | | |
| --- | --- | --- | --- | --- | --- | --- |
| Definition of positive | TST-/IGRA- n (%) | TST+/IGRA+ n (%) | TST-/IGRA+ n (%) | TST+/IGRA- n (%) | Kappa (95% CI) | Concordant (%) |
| ≥ 5 mm | 322 (79.70) | 28 (6.93) | 32 (7.92) | 22 (5.45) | 0.43 (0.31- 0.56) | 86.63 |
| **Agreement between baseline IGRA results and TST results at 3-month follow-up survey** | | | | | | |
| Definition of positive | TST-/IGRA- n (%) | TST+/IGRA+ n (%) | TST-/IGRA+ n (%) | TST+/IGRA- n (%) | Kappa (95% CI) | Concordant (%) |
| Converted* or persistent positive**^†^** (≥ 5 mm) | 298 (73.76) | 45 (11.14) | 15 (3.71) | 46 (11.39) | 0.51 (0.40- 0.61) | 84.90 |

**Abbreviation:** CI, Confidence interval; IGRA, Interferon-γ release assay; TST, Tuberculin skin test; A cutoff value of 0.35 IU/mL was used for IGRA.

*TST conversion was defined as the average diameter of induration ≥ 10 mm at follow-up survey.

**†**Persistent positive was defined as both positive for baseline and follow-up surveys according to the baseline positivity cutoff value.

**
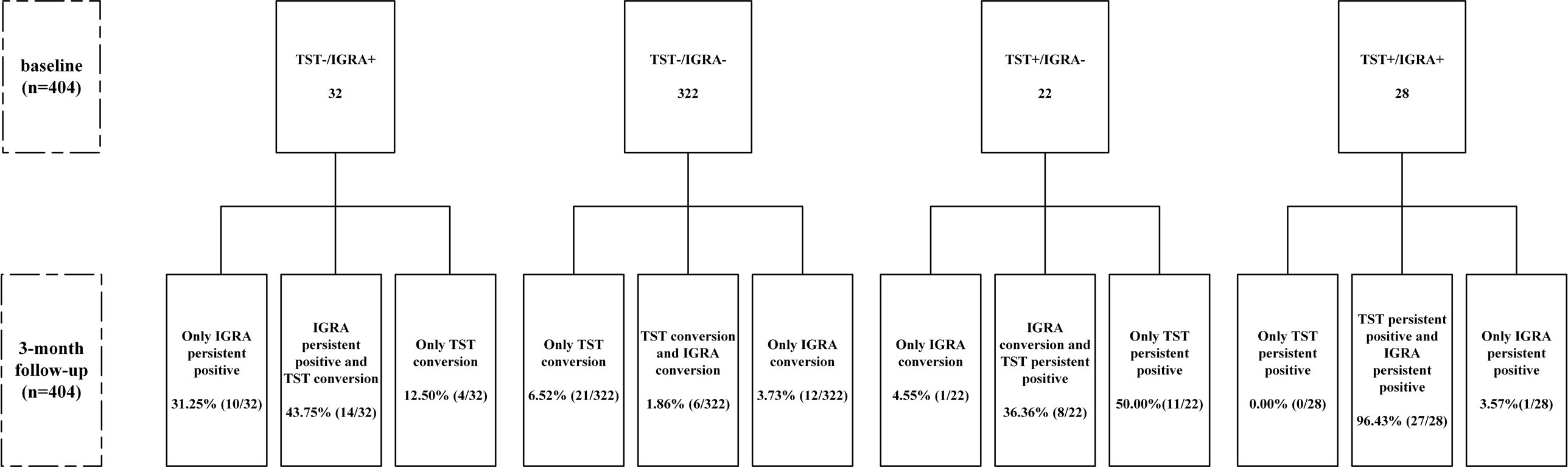
**

**Supplementary figure 1**

**Flow chart of serial testing of TST according to IGRA results.** Stratified analysis according to baseline results of IGRA and TST (using cutoff value of 5mm). IGRA, Interferon-γ release assays; TST, Tuberculin skin test. TST conversion was defined as the average diameter of induration ≥10 mm at follow-up survey. IGRA conversion was defined as the IFN-γ releasing level of TB antigen-Nil (TBAg-Nil) increased from <0.35 IU/ml at baseline survey to ≥0.35 IU/ml at follow-up survey. Persistent positive was defined as both positive for baseline and follow-up surveys according to the baseline positivity cutoff value.


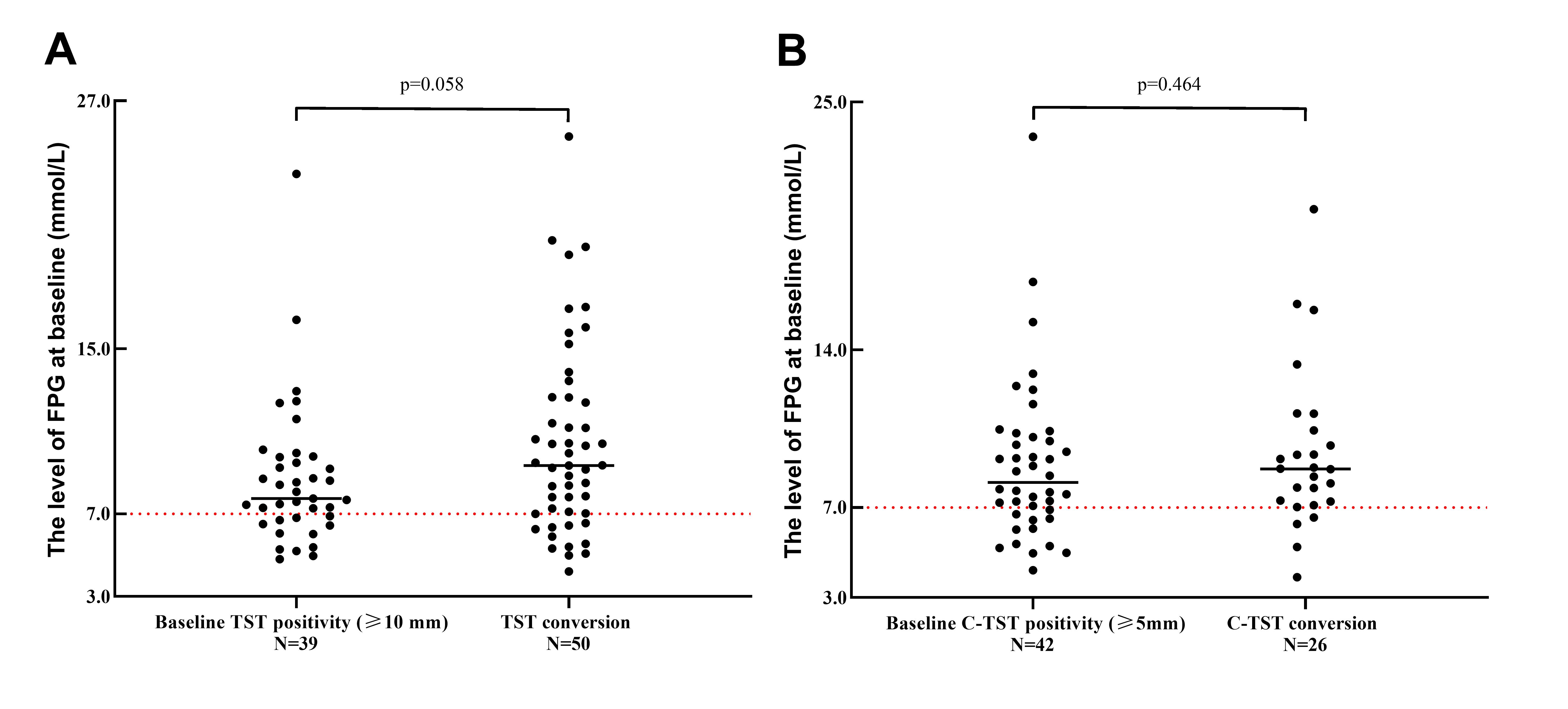


**Supplementary figure 2**

**The level of FPG in the baseline positives and conversions groups of TST and C-TST. (A)** Stratified analysis according to the baseline and follow-up results of TST (using cutoff value of 10 mm); **(B)** Stratified analysis according to the baseline and follow-up results of C-TST (using cutoff value of 5 mm). The differences were tested by Wilcoxon test. C-TST, Creation tuberculin skin test; FPG, Fasting plasma glucose; TST, Tuberculin skin test. TST conversion was defined as the average diameter of induration ≥10 mm for those with baseline survey < 5 mm, or increased ≥ 10 mm when the baseline survey result was between 5 and 10 mm. C-TST conversion was defined as the average diameter of induration and erythema increased from < 5mm at baseline survey to induration or erythema ≥ 5mm at follow-up survey.
